# Editorial: Immunology and inflammation in intracranial aneurysms

**DOI:** 10.3389/fnagi.2022.1046762

**Published:** 2022-10-10

**Authors:** Qingyuan Liu, Bing Zhao, Qin Hu, Shuo Wang, Chengcheng Zhu

**Affiliations:** ^1^Department of Neurosurgery, Beijing Tiantan Hospital, Capital Medical University, Beijing, China; ^2^China National Clinical Research Center for Neurological Diseases, Beijing, China; ^3^School of Medicine and Departments of Neurosurgery, Shanghai Jiao Tong University, Shanghai, China; ^4^Department of Radiology, University of Washington, Seattle, WA, United States

**Keywords:** intracranial aneurysm, immunology, inflammation, biomarker, therapeutic target

Intracranial aneurysms (IAs) are found in ~3% of the American population (Juvela et al., 2008), and 6–7% of the Chinese population (Li et al., 2013), while IA rupture is the main cause of non-traumatic subarachnoid hemorrhage. As a cerebrovascular disease, its initialization and progression are closely related to immunological and inflammatory processes. Recently, more and more studies have revealed that the IA-related immunological and inflammatory factors, or IA-related molecules, could serve as predictors of outcomes of patients with IAs, and these factors could be a treatment candidate for preventing further progression of IAs (Liu et al.; Monsour et al., 2022; Liu et al., 2021). However, few studies have been conducted to investigate the effects of the immune system and inflammation on the progression of IAs. Therefore, this Topic aims to introduce works related to the immunological characteristics of IAs, new biomarkers and models for outcome prediction of patients with IAs, and the potential treatments to prevent IA rupture or growth.

Excitingly, in this Topic, we have selected six excellent works from a bunch of articles. Among them, two studies presented biomarkers to predict the clinical outcomes, and the other four explored the potential pathological characteristics of IAs. Moreover, we believe that these works would provide inspiring insights into the immunological characteristics of IAs, as shown in Figure 1, and more information on precision treatments for IAs.

**Figure 1:**
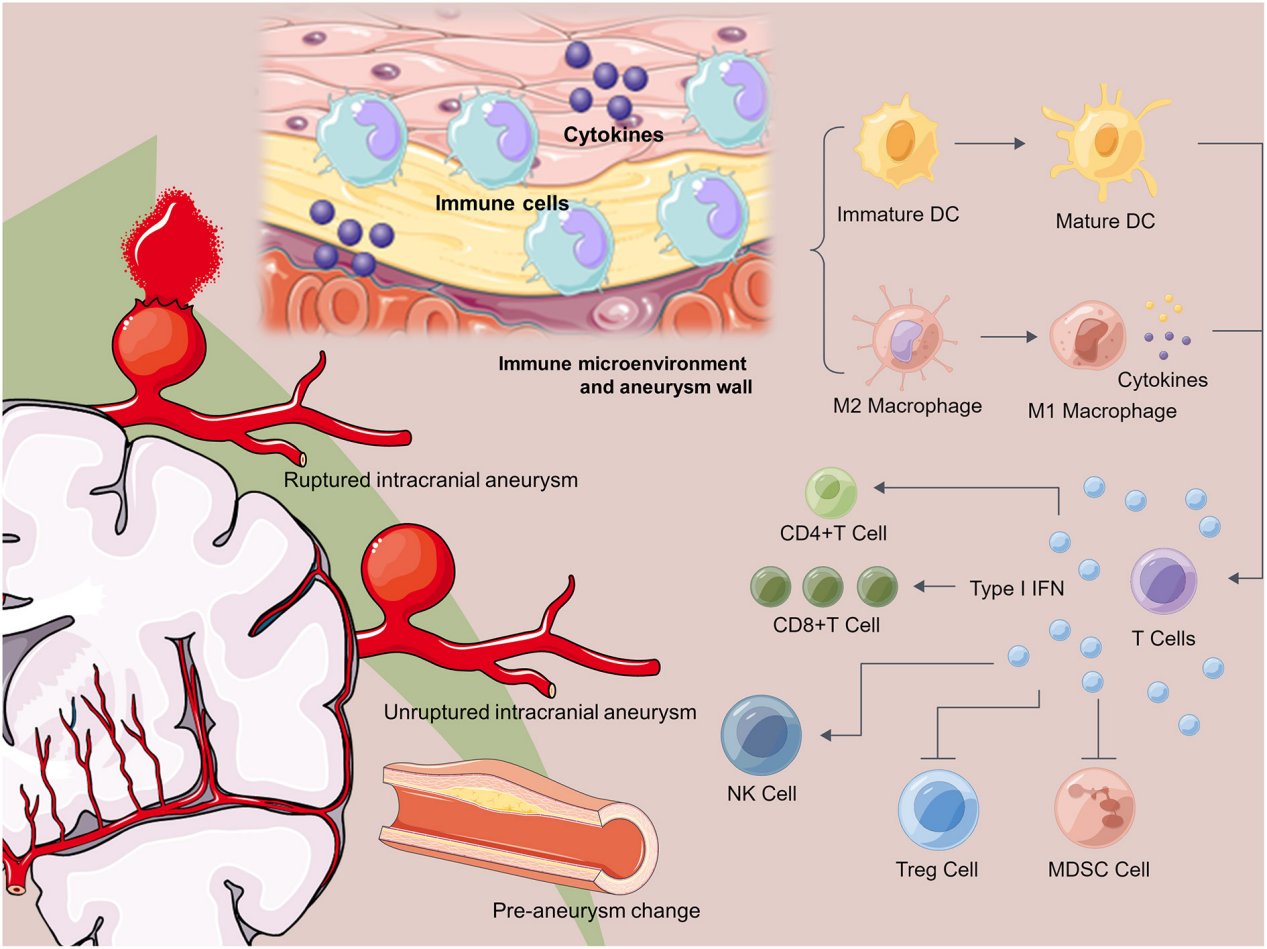
Immunology and inflammation for intracranial aneurysms. The development of intracranial aneurysm includes three stages: pre-aneurysm stage, unruptured intracranial aneurysm and ruptured intracranial aneurysm. Dysregulation of immune microenvironment and inflammation infiltration are the most important pathological characteristics of aneurysm wall. Immune cells and cytokines participate into the dysregulation of immune microenvironment in aneurysm wall.

First of all, I want to introduce the two clinical biomarker studies. Han et al. conducted an interesting multi-center study, which investigated the relationship between haptoglobin phenotypes and neurological outcomes in patients with aneurysmal subarachnoid hemorrhage. By prospectively following up 336 patients, they observed that patients with increased haptoglobin, especially increased haptoglobin 2-2, had a poor 6-month neurological outcome and cognitive impairment. Based on the findings of Han et al., researchers could subsequently move on to develop a quick and efficient biomarker to identify a high risk of poor neurological outcomes for patients with IAs. Another study by Yang et al. explored the relationship between the high risks of IAs and serum levels of cytokines. Cytokines play a key role in the initialization and development of inflammation. In this study, they analyzed 184 serum samples from 184 patients with unruptured IAs and found that increased IL-15 (interleukin 15) and TNF-β (tumor necrosis factor-beta) were correlated to IA progression. The conclusion of this study showed that serum cytokines were potential biomarkers and therapeutic targets for IA progression. These two studies built a connection between the clinical presentation of IAs and their inflammatory responses, which may facilitate the management of patients with IAs.

Among the rest, two studies focused on the pathological and immunological characteristics of IAs. By single-cell RNA sequencing, Wen et al. revealed the correlation between atherosclerosis and blood blister-like aneurysms (BBAs). Macrophages, especially M1 macrophages, are the main inflammatory cells of infiltration into the IA wall. This study showed that the dysregulated genes of macrophages in BBAs were concentrated where the lipid metabolism and formation of atherosclerosis occurred. Furthermore, secreted phosphoprotein 1 (osteopontin) was identified as an important gene to regulate the formation of BBAs. This study is interesting because IA samples are so rare and small in size, so that studies on key IA genes by single-cell RNA sequencing are scarce. In addition, Ge et al. revealed that the dysfunction of circulating immune cells plays a potential role in the development of IAs. In this study, samples of peripheral blood mononuclear cells from 26 IA patients and 20 healthy control patients were analyzed to produce the following results: IA patients had lymphocyte and monocyte overactivation, with increased expression of Toll-like receptor 4 (TLR4), phosphorylated signal transducer and activator of transcription 3 (p-STAT3), and programmed cell death protein 1 (PD-1). This study further proves that immune dysregulation is significant in IA patients.

Finally, the remaining two studies utilized public databases to inspect the immunological characteristics of IA patients. Lu et al. reported the alterations in the immune microenvironment of IAs. They further developed the concept of the score of a pathological feature-derived gene signature, and used the scores in IA diagnosis and rupture risk prediction. And the study by Li et al. revealed that m6A modification might shape the IA microenvironment and participate in IA formation and rupture by regulating the immune infiltration. These two impressive studies also contribute to the discovery of the immunological characteristics of IAs.

Based on a comprehensive framework, this Topic and current studies of IAs still have several limitations. First, mechanisms of IA formation and rupture are still insufficient, while most current studies didn't adopt human IA samples and IA animal models, making their results unreliable. Second, we didn't find relevant content about translational research in terms of IAs when the gap between basic work and clinical practice is remarkable. So, further studies in consideration of clinical scenarios are expected. Third and last, we lack studies on IAs with a multi-omic approach. Multiomics can provide additional information about the mechanisms of IA formation and rupture for researchers and physicians. Due to the aforementioned limitations, many current studies are still in the theoretical stage. Further studies should pay more attention to these three aspects.

## Author contributions

Drafting the article: QL. Critically revising the article: SW, QH, BZ, and CZ. Approving the final version of the manuscript on behalf of all authors: SW. All authors contributed to the article and approved the submitted version.

## Funding

This study was supported by the National Natural Science Foundation of China (Grant No. 82071296) and National Key Research and Development Program of the 14th Five-Year Plan (Grant No. 2021YFC2501100).

## Conflict of interest

The authors declare that the research was conducted in the absence of any commercial or financial relationships that could be construed as a potential conflict of interest.

## Publisher's note

All claims expressed in this article are solely those of the authors and do not necessarily represent those of their affiliated organizations, or those of the publisher, the editors and the reviewers. Any product that may be evaluated in this article, or claim that may be made by its manufacturer, is not guaranteed or endorsed by the publisher.
